# Frequency, Suppressive Capacity, Recruitment and Induction Mechanisms of Regulatory T Cells in Sinonasal Squamous Cell Carcinoma and Nasal Inverted Papilloma

**DOI:** 10.1371/journal.pone.0126463

**Published:** 2015-05-28

**Authors:** Hongfei Lou, Jugao Fang, Pingdong Li, Weiguo Zhou, Yang Wang, Erzhong Fan, Ying Li, Hong Wang, Zhongyan Liu, Lei Xiao, Chengshuo Wang, Luo Zhang

**Affiliations:** 1 Department of Otolaryngology Head and Neck Surgery, Beijing TongRen Hospital, Capital Medical University, Beijing, PR China; 2 Beijing Key Laboratory of nasal diseases, Beijing Institute of Otolaryngology, Beijing, PR China; 3 Sections of Pulmonary & Cardiology, University of Illinois at Chicago, Chicago, Illinois, United States of America; Rutgers - New Jersey Medical School, UNITED STATES

## Abstract

**Background:**

Sinonasal squamous cell carcinoma (SSCC) and nasal inverted papilloma (NIP) represent the predominant type of malignant and benign tumors in sinonasal tract, respectively. CD4^+^CD25^+^Foxp3^+^ natural regulatory T (Treg) cells might play critical role(s) in the suppression of anti-tumor immune response and thus shed light on tumor progression from benign to malignant.

**Objective:**

This study aimed to evaluate the frequency and suppressive capacity of Treg cells in SSCC compared to NIP and further to explore the underlying mechanisms.

**Patients and Methods:**

Frequencies of Treg, Th1 and Th2 cells were evaluated by flow cytometry in tissue homogenate and peripheral blood from 31 SSCC patients, 32 NIP patients and 35 normal controls. Treg cells were tested for regulatory function by co-culture with effector T cells. CCR4 and its ligands, CCL22 and CCL17, were analyzed by flow cytometry and Luminex, respectively. The chemoattractant properties of CCR4/CCL22 and CCR4/CCL17 for Treg cells were assessed using the Boyden chamber technique, to elucidate the potential mechanisms of Treg recruitment in tumor microenvironment. Treg cells induction via TGF-β was assessed with transwells after local CD4^+^Foxp3^+^ T cells were assessed by immunohistochemistry and TGF-β concentration was measured by Luminex.

**Results:**

Tumor-infiltrating Treg cells increased significantly from normal to NIP to SSCC (P ≤ 0.001 for normal vs. NIP and P = 0.004 for NIP vs. SSCC). Significantly elevated frequency and enhanced suppression capacity of circulating Treg cells in SSCC were detected compared to NIP and healthy controls, concomitant with Th1 decrease and Th2 increase. Apparently increased CCL22 attracted CCR4-expressing Treg cells to tumor microenvironment in SSCC, compared to NIP. SSCC produced significantly more TGF-β than NIP and thus possessed greater potential for Treg cell induction.

**Conclusion:**

Frequency and suppressive capacity of Treg cells enhanced with progression of malignancy from NIP to SSCC. Circulating Treg cells were recruited to tumor tissue via CCR4/CCL22 signalling, whereas tumor-synthesised TGF-β contributed to induction of peripheral Treg cells.

## Introduction

Sinonasal squamous cell carcinoma (SSCC) is the predominant type of solid cancer in sinonasal tract, with 30.2% of the patients demonstrating a 5-year survival rate[1], while nasal inverted papilloma (NIP); one of the most common benign sinonasal tumors[2] exhibiting malignant behavior including recurrence tendency, destructive capability, and propensity to malignancy; is concomitantly diagnosed in 1.7%-56% of patients with SSCC[3,4,5,6].

Several studies have indicated that the elevation of CD4^+^CD25^+^Foxp3^+^ natural regulatory T (nTreg) cells in a variety of malignancies might contribute to tumor progression by evading immune recognition and promoting an immunosuppressive environment, which were associated with poorer prognosis and reduced survival[7–12]. Accumulating evidence has further shown that head and neck squamous cell carcinoma (HNSCC) patients harbour increased levels of nTreg cells with greater suppressive activity, compared to healthy controls[13,14,15,16]. However, while some studies have linked higher Treg cells levels to advanced tumor stage and nodal metastasis[15–17] in HNSCC, others have provided conflicting results[10,14]. Additionally, our studies have indicated that the frequency, rather than suppressive capacity, of nTreg in local NIP tumors was significantly increased relative to normal controls[18]. To date, however, neither infiltrating nor circulating Treg cells have been compared between SSCC and NIP. Furthermore, the mechanism(s) of Treg cells enrichment in patients with tumors is not well understood. Some studies have demonstrated that two ligands of CC-chemokine receptor 4 (CCR4), CCL22 and CCL17, are strongly correlated with the increased infiltration of Treg cells in ovarian tumors[7], esophageal squamous cell carcinoma[19] and gastric cancer[20]. Other studies have reported tumor cells to serve as a source of TGF-β, which is required for the induction and maintenance of Treg cells, leading to elevated Treg cells in peripheral blood[21,22].

The aim of this study was thus to firstly evaluate the frequencies of CD4^+^CD25^+^Foxp3^+^ Treg cells in tumor tissue as well as in peripheral blood in patients with NIP and SSCC. Secondly, the study aimed to compare the suppressive capacity of circulating Treg cells in the patients with malignant progression from normal to NIP to SSCC. Furthermore, we also explored the putative mechanisms underlying Treg cells recruitment and induction in patients with NIP and SSCC.

## Materials and Methods

### Patients and specimens

Overall, 31 SSCC patients (19 males and 12 females; age range, 32–77 years; all T_1-2_N_0_M_0_) and 32 NIP patients (18 males and 14 females; age range, 30–72 years) were recruited from Beijing TongRen Hospital, between June 2009 and January 2013. Tumor specimens or normal nasal mucosa samples were collected using a standard endoscopic technique and venous blood was simultaneously drawn from each subject into a heparin-treated vacuum test tube simultaneously. The study was approved by the Medical Ethics Committee of Beijing TongRen Hospital and a written informed consent was obtained from each participant prior to entry into the study.

### Flow cytometric analysis

Peripheral blood mononuclear cells (PBMCs) were isolated using Ficoll-Plaque Plus density gradient centrifugation (Amersham Biosciences, NJ, USA). Fresh tissues were washed and cut into small fragments, prior to homogenization by tissue disaggregation vessels (BD Medimachine System). The cells in each sample were adjusted to a concentration of 2 × 10^6^ cells/mL, and 0.5 mL cell suspension stimulated with 2μL Leukocyte Activation Cocktail and BD GolgiplugTM (BD Pharmingen, San Diego, CA, USA) for 4 hours.

T cell subsets were phenotyped in isolated PBMCs or tissue cells by flow cytometry (FACSAria flow cytometer, BD Biosciences, NJ, USA) according to manufacturer’s instructions. The cells were labelled with specific monoclonal antibodies; including anti-CD4-PerCP, anti-CD25-PE, anti- Foxp3-FITC, anti-IL-4-PE and anti-IFN-γ-FITC (all from BD Biosciences, NJ, USA). T cell subsets were selected for detailed phenotypic analysis as follow: (1) Th1 cells: IFN-γ^+^IL-4^-^ CD4^+^ T cells; (2) Th2 cells: IFN-γ^-^IL-4^+^ CD4^+^T cells; (3) Treg cells: CD4^+^CD25^+^Foxp3^+^ T cells. A minimum of 10^6^ cells per staining were assessed, with at least 10^5^ events being measured. To measure CCRs in peripheral Treg cells, anti-CCR3, anti-CCR4, anti-CCR5 and anti-CCR8 (R & D Systems, Mineapolis, MN, USA) were used to evaluate corresponding receptor expression in circulating Treg cells of 3 groups.

### Purification of Treg cells (CD4^+^CD25^+^ CD127^-^ T cells)

For additional functional analysis, we obtained 50 mL heparinised peripheral blood from each of 7 SSCC patients, 6 NIP patients and 6 healthy controls. CD4^+^ T cells were isolated from the PBMCs using a human regulatory T cell isolation kit (MiltenyiBiotec, Germany). CD4^+^ T cells were sorted into 2 populations based on whether or not they expressed CD25 and CD127: CD4^+^CD25^+^CD127^-^ (Treg cells) and CD4^+^CD25^-^CD127^+^ (Effector T cells) by FACS.

### Functional analysis of CD4^+^CD25^+^CD127^-^ T cells in PBMCs

The suppressive capacity of Treg cells was determined by the cytokines synthesised on activation. The isolated CD4^+^CD25^-^ T cells were cultured alone or after mixing with CD4^+^CD25^+^CD127^-^ T cells (in a ratio of 2:1) in a final volume of 200 μl RPMI 1640 medium containing 5% FBS. The cells were cultured and activated using a 96-well anti-CD3 plate (BD bioscience, San Jose, CA, USA) plus anti-CD28 (5μg/mL) for 72 hours. Triplicate medium wells were included as negative controls. At day 3, 100 μl of each supernatant were collected and stored for analysis of Th1 and Th2 cytokine (IFN-γ and IL-4, respectively) by ELISA (R&D Systems, Minneapolis, USA).

### Fluorescent Immunohistochemistry analysis

Treg cell infiltration in local tissue was quantified by Immunohistochemistry. Tumor specimens and normal nasal mucosa were fixed, embedded, and sectioned at 5μm thickness. 15 samples each from SSCC, NIP and normal control subjects were assessed by fluorescent immunohistochemistry, using rabbit-anti-CD4 (staining red) and mouse-anti-Foxp3 (staining green) (both from abcam, UK) to identify Treg cells. The stained sections were observed by two independent physicians, blinded to the clinical diagnosis of the patients, using a fluorescence microscope. All samples were assessed at x400 magnification and double-stained cells CD4^+^Foxp3^+^ Treg cells were counted in10 randomly selected fields per sample. The results were expressed as the mean number of CD4^+^Foxp3^+^ Treg cells in 10 fields.

### Measurement of CCL22, CCL17 and TGF-β in local tissue

To explore the putative mechanisms of Treg recruitment and induction in sinonasal tumors, we evaluated CCL22, CCL17 and TGF-β concentrations in the local tissue. Ten samples from each group were weighed and a total of 1.0mL PBS supplemented with 0.05% tween 20 (Sigma-Aldrich, St Louis, Mo) and 1% protease inhibitor cocktail (Sigma-Aldrich, St Louis, Mo) was added for every 100 mg of tissue in each sample. All samples were homogenized using a standard bench-top homogenizer (Polytron PT 2100, Kinematica, Switzerland) at 1000 rpm for 5 minutes and the homogenates were centrifuged at 1500 ×g for 10 minutes at 4°C.The supernatants were collected and stored at -80°C until further analysis.CCL22, CCL17 and TGF-β in tissue homogenates were assessed using Fluorokine MAP Multiplex Kits and analyzed on a Luminex 100 analyzer (Luminex 100 System, Austin, TX, USA).

### Treg cell chemotaxis assay

Tumor-induced chemotaxis of Treg cells was assessed using 5μm pore size Transwell chambers (Millipore Corp., Billerica, USA). Briefly, Treg cells were isolated from peripheral blood of healthy individuals and after suspension at a concentration of 5×10^4^ cells in a final volume of 200μl DMEM containing 10% FBS, were added to the upper chambers in Transwells. Primary tumor cell culture supernatants (SSCC or NIP) in 1.0ml medium were added to the lower chambers and cultured for 24 hours at 37°C in a 5% CO_2_ in air atmosphere. DMEM containing 10% FBS was used as control medium in the lower chambers. After the end of 24hours’ incubation, the Treg cells that had migrated from the upper to the lower chamber were quantified by flow cytometry. To determine whether CCL22 or CCL17 was responsible for the chemotaxis/recruitment of regulatory the T cells, either 500 ng/mL anti-CCL22or 100 ng/mL anti-CCL17 mAb (both from R&D Systems, Minneapolis, USA) was added to the culture supernatants in the lower chambers, and the cultures processed as above after culture for 24 hours at 37°C in a 5% CO_2_ in air atmosphere.

### Treg cells induction via TGF-β in vitro

Circulating PBMCs from healthy individuals were co-cultured with tumor cells to assess peripheral Treg cell induction via tumor-synthesised TGF-β. Baseline Treg cells in PBMCs were counted by FACS before culture and then 10^6^ isolated PBMCs in 200 μl of medium (DMEM+10% FBS) were seeded on to each Transwell insert (Corning Incorporated., 0.4μm pore size). 0.1g tissue (SSCC, NIP and normal nasal mucosa respectively) was placed into the bottom well, with or without 20μM SB431542 (an inhibitor of the type I TGF-β receptor kinase) in 1ml medium, and cultured with the insert at 37°C in a 5% CO2 in air atmosphere. After 5 days, the medium was collected from the insert well and assessed again for the number of Treg cells as above. Appropriate control sets were prepared with DMEM+10% FBS as negative control and TGF-β (5ng/mL) as positive control.

Any increase in the number of Treg cells from baseline was expressed as inducted Treg cells. Induction capability was expressed as induction ratio, which represented the fold increase/decrease compared to the inducted Treg cells in DMEM+10% FBS. Induction of Treg cells in DMEM+10% FBS was used to normalize the results obtained in other groups.

### Statistical analysis

SPSS version 19.0 (IBM Corp., Armonk, NY) was used for statistical analysis. The Student’s unpaired *t*-test or the Mann-Whitney U-test was used for independent data sets. Additionally, differences between related data sets were analyzed using the student’s paired *t*-test and Wilcoxon signed rank test for normally and not normally distributed data, respectively. Values were considered significant when *P* < 0.05.

## Results

### Proportions of Treg, Th1 and Th2 cells in tumor microenvironment

**Fig 1:**
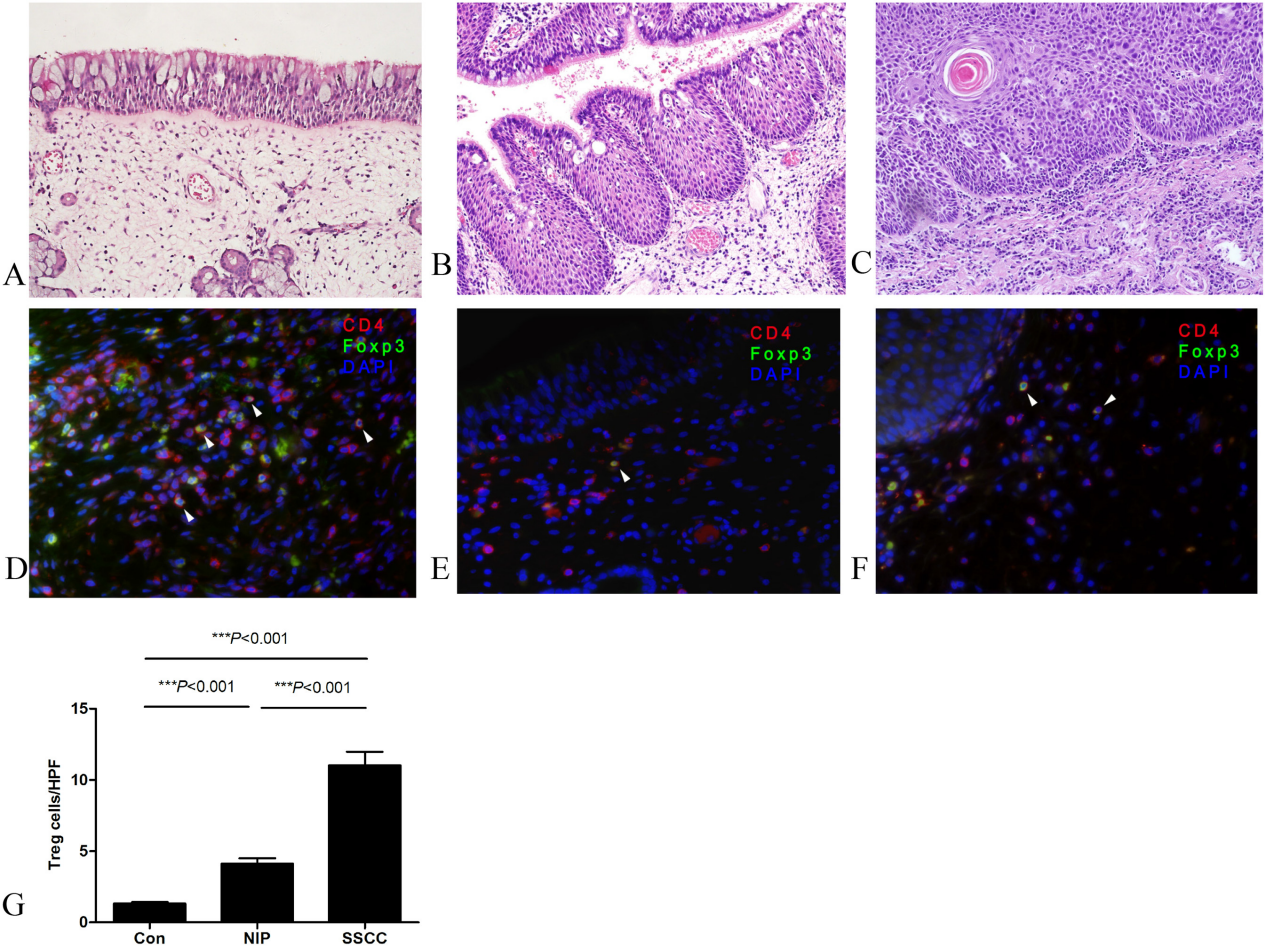
CD4^+^FoxP3^+^ Treg cells (white arrows) in local tissue were determined by fluorescent immunohistochemistry (CD4, green; Foxp3, red; DAPI for nuclei, blue). Representative H & E staining at 200x magnification for Con (A), NIP (B) and SSCC (C) and fluorescent immunohistochemistry stain at 400x magnification for Con (D), NIP (E) and SSCC (F) respectively. (G) Quantification of infiltrating Treg cells in three groups (n = 15). Con, normal control; NIP, nasal inverted papilloma; SSCC, sinonasal squamous cell carcinoma.

The diagnosis of SSCC and NIP was confirmed by histological evidence showing typical characteristics of the respective condition (Fig 1A–1C). Tumor-infiltrating Treg cells were evaluated first by fluorescent immunohistochemistry and flow cytometry. Representative flow cytometry results of Treg cells are shown in supplemental S1E Fig.

**Fig 2:**
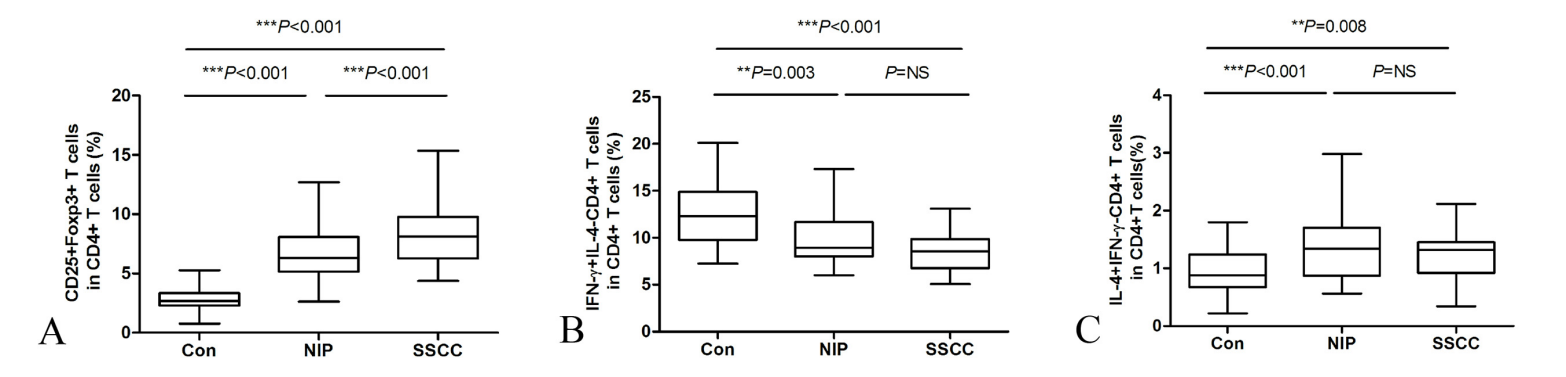
Tumor-infiltrating Treg (A), Th1 (B) and Th2 (C) cells were assessed by flow cytometry. n = 31 for Con, n = 32 for NIP, and n = 35 for SSCC.

Immunohistochemical staining demonstrated that CD4^+^Foxp3^+^ Treg cells were significantly increased with malignant progression from normal to NIP to SSCC (Fig 1D–1G). The increase in the proportion of infiltrating Treg cells was confirmed by flow cytometric analysis (Fig 2A).

The frequency of Th1 cells in normal nasal mucosa was found to be greater compared to not only NIP, but also compared to SSCC (Fig 2B). In contrast, the frequency of Th2 cells were significantly increased in NIP and SSCC compared to normal controls (Fig 2C). The numbers of either Th1 or Th2 cells in NIP and SSCC were not significantly different (Fig 2B and 2C).

### Prevalence of Treg, Th1, and Th2 cells in peripheral circulation

**Fig 3:**
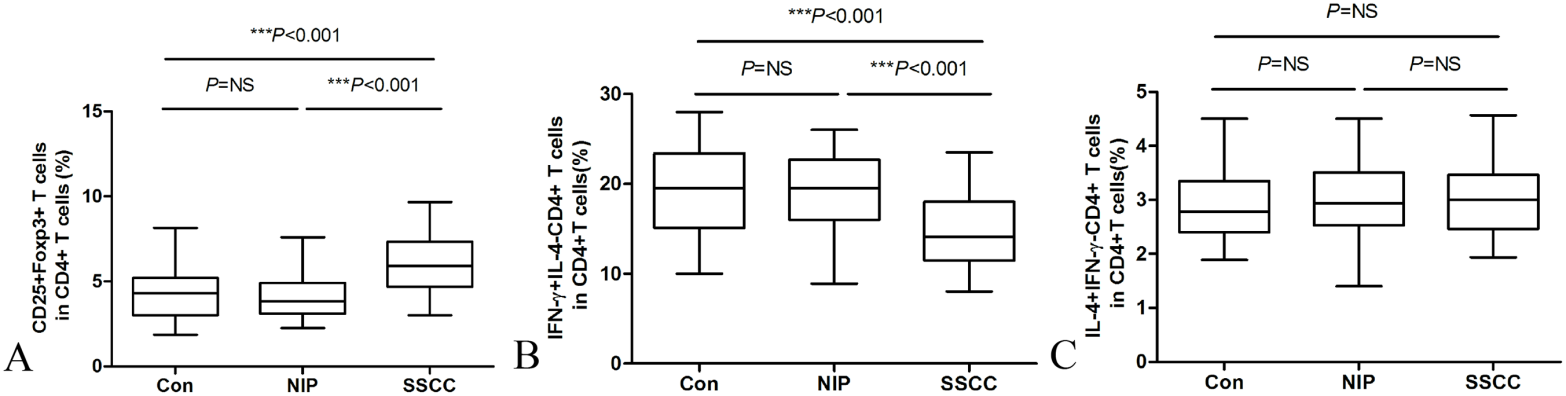
Circulating Treg (A), Th1(B) and Th2 (C) cells were evaluated by flow cytometry. n = 31 for Con, n = 32 for NIP, and n = 35 for SSCC.

SSCC patients had significantly elevated Treg cells in peripheral blood compared with NIP patients (*P* < 0.001) and healthy controls (*P*< 0.001) (Fig 3A).In contrast, the Th1 cell population was significantly lower in the peripheral circulation in SSCC patients compared to the other two groups (both *P*< 0.001) (Fig 3B). Both peripheral Treg and Th1 cells in patients with NIP were comparable to that in control groups. Comparison of the frequencies of Th2 cells in peripheral circulation indicated that these were not significantly different between any of the groups (Fig 3C).

### Expression of CCL22/CCL17 inducing Treg cells migration via CCR4

**Fig 4:**
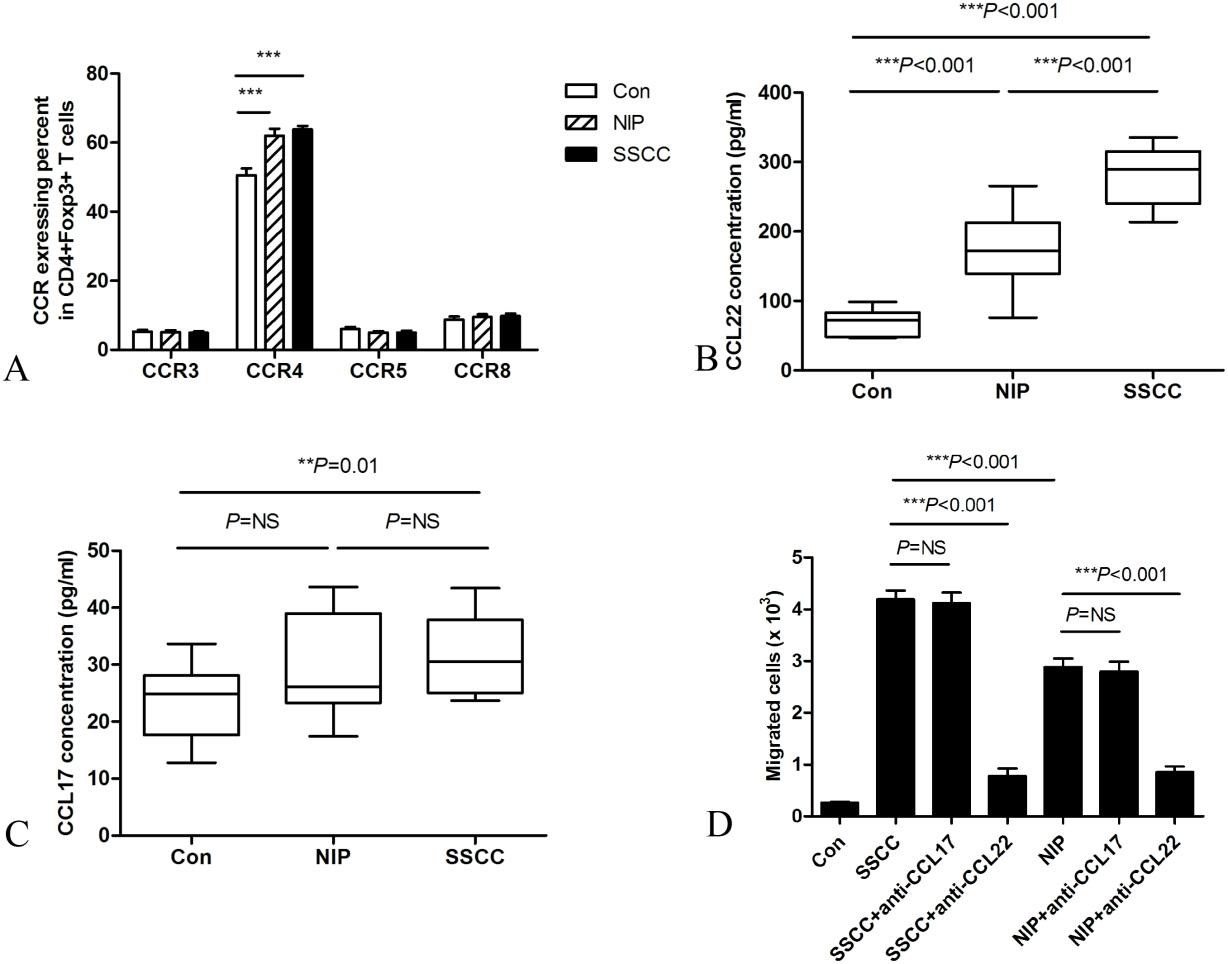
Expression of CCRs in circulating-Treg cells (A) and local concentration of CCR4 ligands, CCL22 (B) and CCL17 (C), were assessed by flow cytometry and Luminex, respectively. Tumor culture supernatants from 6 SSCC and 6 NIP were used to stimulate chemotaxis of peripheral Treg cells isolated from healthy adults (D). ***P<0.001 in Fig 4A.

The expression of CCR4 was more pronounced than expression of CCR3, CCR5, and CCR8 in circulating Treg cells (Fig 4A);with a significantly lower number of Treg cells in normal controls (50.52%) expressing this chemokine receptor than Treg cells in NIP (62.02%)or SSCC (63.82%) (Fig 4A).The expression of ligand CCL22 was increased with invasive progression from normal to NIP to SSCC in local tumor (Fig 4B). In contrast, although CCL17 was expressed more abundantly in SSCC than in healthy control (Fig 4C), this was comparable between NIP and control, and also much lower compared to expression of CCL22 in the same sample.

Assessment of tumor-induced chemotaxis of Treg cells also demonstrated that although supernatants from both SSCC and NIP induced a significantly greater chemoattractant response compared to control medium, the SSCC-induced effect was also significantly greater compared to NIP-induced effect on Treg cells (Fig 4D). Blocking experiments further demonstrated that anti-CCL22, but not anti-CCL17, significantly suppressed the chemotaxis of Treg cells in response to supernatants from both SSCC and NIP.

### Suppressive capacity of circulating Treg cells

Further studies were performed to assess the functional activity of circulating CD4^+^CD25^+^CD127^-^ Treg cells from patients with SSCC and NIP, compared to healthy donors, by investigating their ability to suppress autologous CD4^+^CD25^-^ T cells upon stimulation with CD3/ CD28.

**Fig 5:**
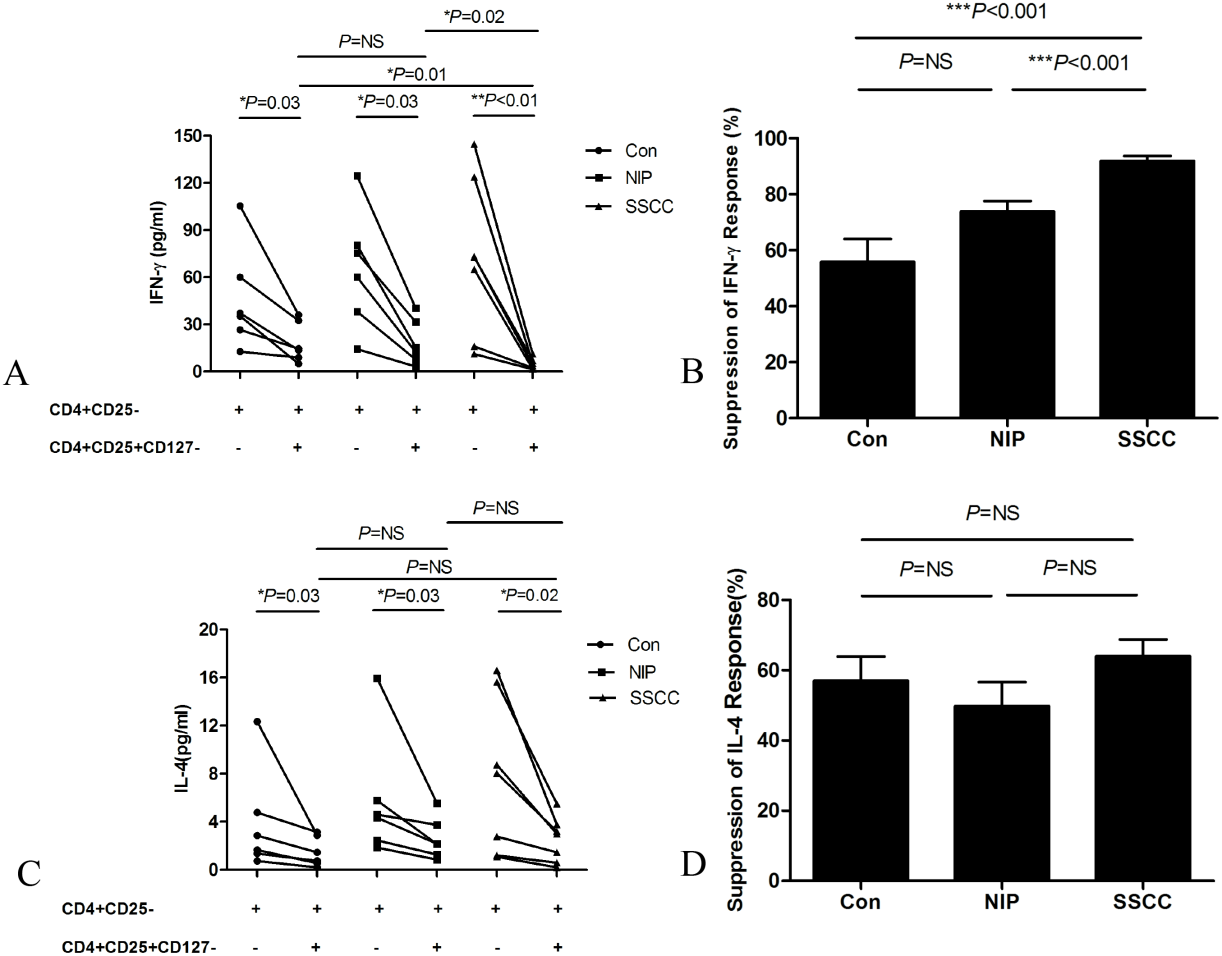
The suppressive capacity of circulating CD4^+^CD25^+^CD127^-^ Treg cells cells on CD4^+^CD25^-^ cells was examined in SSCC patients (n = 7), NIP patients (n = 6) and healthy controls (n = 6). (A) IFN-γ production, (B) suppression percentage of IFN-γ response, (C) IL-4 production, (D) suppression percentage of IL-4 response were shown.

As indicated in Fig 5A, Th1 cytokine (IFN-γ) production in CD4^+^CD25^-^ T cells in response to CD3/CD28 stimulation was significantly inhibited by CD4^+^CD25^+^CD127^-^ Treg cells in three cohorts; with the suppression of IFN-γ by Treg cells being more remarkable in patients with SSCC than in NIP patients or healthy controls (Fig 5A and 5B). However, there was no significant difference in IFN-γ suppression in NIP patients or healthy controls, and complemented the findings for theTh1 cell population in the peripheral circulation (Fig 3B).

Evaluation of Th2 cytokine (IL-4) production after co-culture with or without Treg cells indicated that CD4^+^CD25^+^CD127^-^ Treg cells obtained from all three groups significantly suppressed IL-4 produced by CD4^+^CD25^-^ T cells (Fig 5C). However, the suppressive capacity of Treg cells from the three groups was not significantly different (Fig 5D), and complemented the findings for the Th2cell population in peripheral blood (Fig 3C).

### Peripheral Treg cells induction via TGF -β produced by tumors

**Fig 6:**
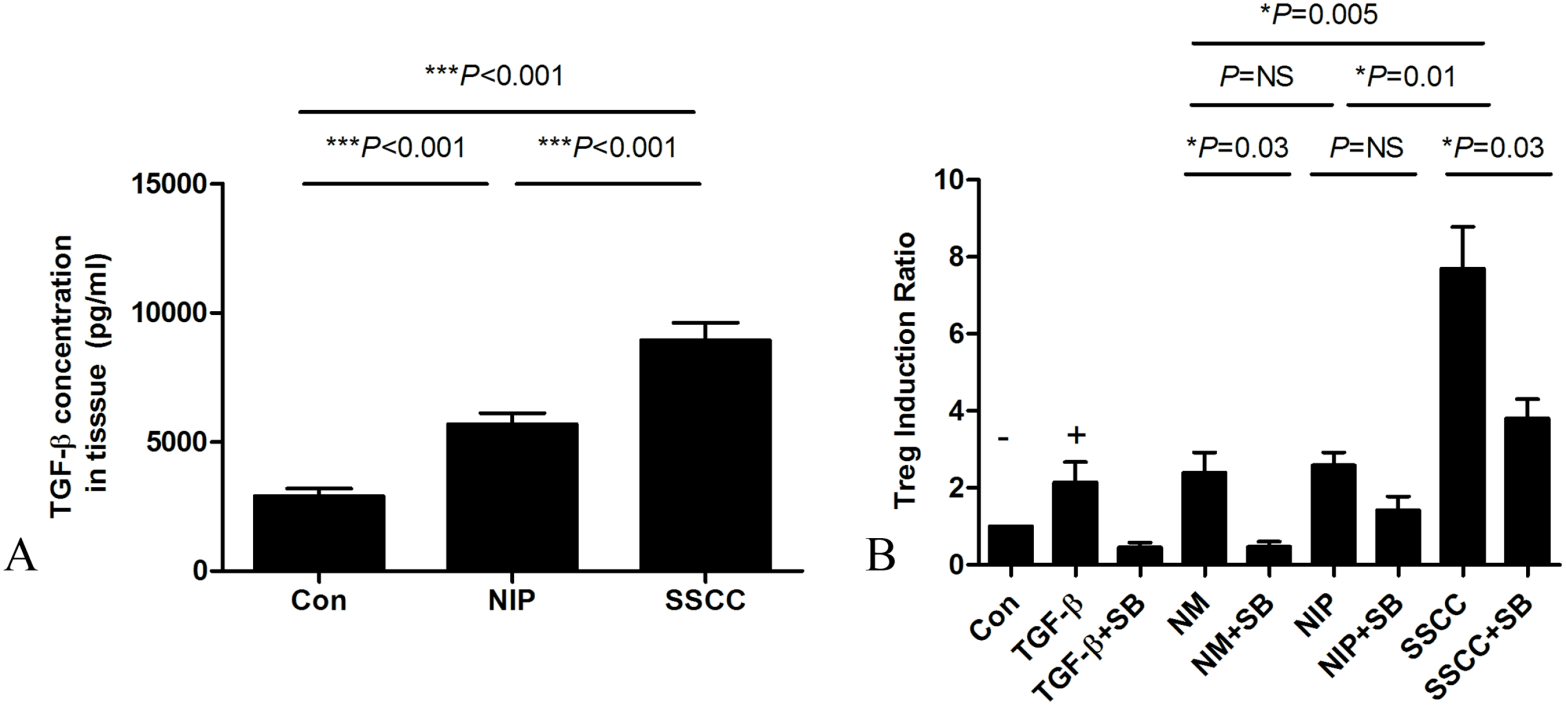
TGF-β in local tissue induced Treg cells in peripheral blood. (A) Tissue TGF-β concentration was analyzed by Luminex (n = 10). (B) Treg cells were induced in transwell. Results are shown as mean ± SEM. Con, DMEM+10%FBS; SB, an inhibitor of the type I TGF-β receptor kinase SB431542; NM, normal mucosa; NIP, nasal inverted papilloma; SSCC, sinonasal squamous cell carcinoma. n = 4 independent experiments.

Both NIP and SSCC produced significantly greater concentrations of TGF-β compared to the normal mucosa, however, the amount of TGF-β produced by SSCC was also significantly greater than that produced by NIP(Fig 6A). Assessment of the induction of Treg cells in transwell indicated that this was significantly increased by all tissues compared to the negative control. Moreover, the induction of Treg cells was significantly greater with SSCC, compared to both NIP and normal mucosa, which were not significantly different (Fig 6B). However, addition of SB431542 attenuated the TGF-β and tissue-induced Treg induction (Fig 6B).

## Discussion

Emerging evidence supports the notion that Treg population plays a critical role in the suppression of anti-tumor immune response and thus contributes to cancer progression. However, to date there is no report in the literature comparing Treg cells between malignant and benign tumors in sinonasal tract. To expand our understanding on the role of Treg cells in this regard, in the present study we investigated both the frequency and function of circulating Treg cells, as well as tumor-infiltrating Treg cells, in patients with SSCC or NIP and compared these with nasal mucosal tissue from healthy control subjects. Our data indicated that there was a significantly increased Treg population with enhanced suppression capacity in both local tumor and peripheral blood in SSCC patients, compared to NIP patients and control subjects. Furthermore, our study demonstrated that the malignant tumor produced significantly greater amounts of TGF-β and also led to a significantly greater induction of Treg cells in transwell, compared to the non-malignant tumor and normal nasal mucosa. Collectively, these findings suggest that both peripheral and local tumor infiltrating Treg cells are likely to play a regulatory role in tandem in SSCC, and that the malignant tumor acquires an advantage by induction of circulating Treg cells via TGF-β production and invasion of immune surveillance.

Treg cells have been shown to be present in increased proportions and exert enhanced function in the peripheral circulation of patients with various malignancies[7,10,11,23], which may possibly be predictive of significantly reduced patient survival. Our finding for significantly elevated Treg cells in peripheral blood of SSCC patients compared with NIP patients and healthy individuals is in accordance with the findings of other studies[10,14,15,24]. Our study has additionally demonstrated that Treg cells obtained from SSCC exhibited a significantly greater suppression on effector T cells compared to Treg cells from NIP. Collectively, these results suggest that there may be a relationship between not only the quantity but also the “quality” of Treg cells and the malignancy of a tumor. Furthermore, as an elevation in circulating Treg cells in SSCC, but not NIP, appears to be concomitant with a significant suppressive effect on Th1 cells, these findings suggest that malignant sinonasal tumors may induce a profound systemic response. In contrast, as the increase in tumor-infiltrating Treg cells in NIP was accompanied with decreased Th1 and elevated Th2 infiltration, these findings suggest that in NIP, Treg cells may be involved in local, rather than systemic tumor immunity.

The crucial role of CCR4 expression by Treg cells in the development of immune tolerance has been demonstrated in some studies[25,26]. We observed that CCR4-expressing Treg cells were significantly higher in SSCC and NIP, compared to healthy controls. In accordance with Schott’s findings[14] for patients with head and neck squamous cell carcinoma (HNSCC), we also found significantly elevated level of the CCR4 ligand, CCL22, in SSCC patients compared to healthy subjects. A mirrored trend with significance was also revealed in NIP patients as well. Although CCL17 was up-regulated in SSCC relative to controls, its level was comparatively lower compared to CCL22 in the same patient. In accordance with this finding, Toulza and colleagues[27] have previously demonstrated that the concentration of CCL22 in adult T-cell leukemia/lymphoma patients was abnormally high and correlated with the frequency of CD4^+^FoxP3^+^ cells. Moreover, CCL22 enhanced the migration and survival of CD4^+^Foxp3^+^ cells in vitro. In contrast, little or no CCL17 was detected in the plasma of these patients. Similarly, Gobert and colleagues[28] found a strong correlation between infiltrating Foxp3^+^ Treg cells and CCL22, but not with CCL17, in breast tumor. In the present study we investigated the effects of tumor-induced chemotaxis of Treg cells in vitro and the potential role of CCL22 or CCL17 in this process, and demonstrated that SSCC and NIP both significantly increased the chemotaxis of Treg cells. Indeed, SSCC-induced chemotaxis of Treg cells was significantly greater than NIP-induced chemotaxis and mirrored the pattern of Treg cell accumulation noted in the tumor microenvironments. Moreover, this study demonstrated that both SSCC- and NIP-induced chemotaxis of Treg cells in vitro was significantly suppressed by anti-CCL22, but not anti-CCL17. These findings are in accordance with the findings of Curiel and colleagues [7], who also demonstrated significant chemotaxis of Treg cells in response to malignant ascites in ovarian carcinoma, and inhibition of chemotaxis in vitro by anti-CCL22, but not anti-CCL17. Collectively, these findings provide compelling evidence, which suggests that higher accumulation of Treg cells in local tumor maybe a consequence of elevated levels of CCL22 in the tumor microenvironment attracting CCR4-expressing Treg cells.

Th1/Th2 cytokine profile is frequently correlated with human neoplastic diseases. Tumor-infiltrating T cells in SSCC and NIP were inclined to a Th2-biased profile in our study, which has also been highlighted in circulation of HNSCC and NIP patients by other authors[13,18,29]. Bose and colleagues[13] and Lathers and colleagues[30]have additionally demonstrated decreased secretion of Th1 cytokines in HNSCC patients, in accordance with the findings from the present study. Cancer cells promote the production of IL-4 and down-regulate the level of IFN-γ in cancer-encountered T cells[31]. Additionally, Th2 cytokines (IL-4 in particular) have been shown to temporarily suppress IFN-γ production[13,29]. This Th2 bias could prevent potentially beneficial anti-tumor immune response because IFN-γ possesses potent cytotoxic properties towards tumor. Indeed, it has been demonstrated that IFN-γ reduced the expression of CCL22 in HNSCC[14]. Overall, the general finding that attenuation of IFN-γ facilitates the production of CCL22 and results in recruitment of Treg cells to tumor sites, in turn resulting in additional inhibition on IFN-γ, leads us to speculate that the cyclic interaction between IFN-γ, CCL22, and Treg cells may comprise a positive feed-back mechanism for increasing the survival and malignancy of tumor cells.

Currently, the precise mechanism/s underlying an increase of peripheral Treg cells in SSCC remains unclear. A recent study demonstrated that hypoxia up-regulated the expression of TGF-β1, thereby inducing Treg increase in gastric cancer[32]. Our finding of higher TGF-β level in a tumor-related microenvironment is in accordance with this finding. To test the hypothesis that tumor burden is partially responsible for circulating Treg elevation via TGF-β, circulating PBMCs from healthy individuals were co-cultured with tumor tissue with or without SB431542, a TGF-β receptor inhibitor. Our results suggested that the induction of Treg cells cultured with malignant tumor was significantly higher than that cultured with NIP tissue or normal mucosa, and corresponded with TGF-β expression in the different tissues. However, addition of SB431542to the culture medium attenuated, but not completely blocked all tissue-induced Treg cells cell, suggesting that apart from TGF-β other cytokines were also likely to be involved.

In conclusion our study had demonstrated that both tumor-infiltrating and circulating Treg populations are significantly increased in SSCC compared to NIP or normal subjects and exhibit a higher level of suppressive activity on effector T cells. Treg cells induction and recruitment to the local tumor tissue via CCR4/CCL22 signalling and tumor-derived TGF-β suggests that there is cross-talk between Treg cells and the tumor, which possibly leads to increased survival and malignancy of the tumor. It is noteworthy that accumulation of functional Treg cells in the peripheral blood and/or at the tumor site of patients may be a biomarker of progression from NIP to SSCC, which needs further exploration. Moreover, the present findings provide evidence for potential design of a novel immunotherapeutic strategy for specifically targeting the tumor-protecting role of Treg cells in patients with sinonasal squamous cell carcinoma. Thus, elimination of Treg cells within the tumor microenvironment, for example by local micro-injection of anti-TGF-β or anti-CCL22 monoclonal antibodies might be an effective therapeutic approach against SSCC. Similarly, targeted therapy with chemical agents such SB431542 and LY2157299, which inhibit TGF-β signalling, may also provide an alternative approach.

## Supporting Information

S1 Fig Treg cells were evaluated by flow cytometry. Scatter dots in the right upper quadrant represent CD4+CD25+Foxp3+ T cells (Treg cells) in (A) Con, (B) NIP, and (C) SSCC, respectively. Values in dot plots indicate percentages of Treg cells in total CD4+ T cells.
(TIF)
