# Supplementary figures and images for: Frequency, Suppressive Capacity, Recruitment and Induction Mechanisms of Regulatory T Cells in Sinonasal Squamous Cell Carcinoma and Nasal Inverted Papilloma
Source: PLoS One. 2015 May 28;10(5):e0126463. doi: 10.1371/journal.pone.0126463 (PMC4447263; doi:10.1371/journal.pone.0126463)

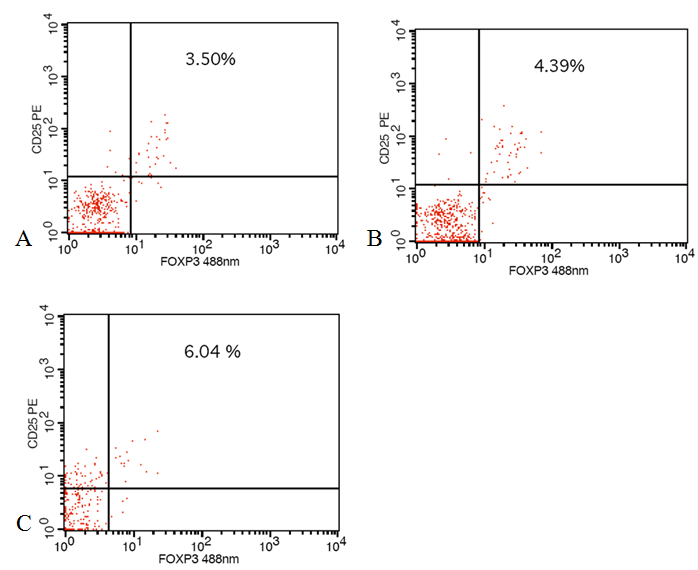

Supplement: S1 Fig — Scatter dots in the right upper quadrant represent CD4+CD25+Foxp3+ T cells (Treg cells) in (A) Con, (B) NIP, and (C) SSCC, respectively. Values in dot plots indicate percentages of Treg cells in total CD4+ T cells. (TIF) [file pone.0126463.s001.tif]
